# Paris Items

**Published:** 1868-03-01

**Authors:** 


### *Paris Items.*

From a private letter we scissor these bits of medical gossip: "Prof. Richet, one of the best surgeons in Paris, attempted to perform ovariotomy on a woman thirty-five years old. After working an hour, he found so many adhesions that he was obliged to discontinue the operation, dress the wound, and send the woman back to bed, where she will, undoubtedly, soon die. Truly the French surgeons have no success with this part of surgery. Either they can not do it properly, or else their subjects are bad, for they invariably die. Prof. Nelaton has discontinued his hospital service to be named, as it is said, Senator. He has been in great favor at court for some time, and probably Napoleon desires to honor him as much as his physician, Dr. Comeau, who, you are aware, has attained the Senatorial honor. Many changes have been made in the medical school within the last few months. Many of the venerable old professors having either died or been put *hors de combat*, to be replaced by a younger generation. Some of the newly-appointed, for young men, have already obtained much celebrity."

We regret to hear this report of M. Nelaton, after his previous dignified refusal to wade in the mire of politics. Alas for the great surgeon who

> "Born to a universe, narrows his mind,
> And to a platform gives up what was meant for mankind."

When a doctor meddles with politics, he should cease finding fault with parsons for meddling with physic. Which reminds us of the current rumor that *the* apostle of medical reform is, to the detriment of his Æsculapian robes, putting himself in training for the lower (if possible) house in Congress. It can no longer be said, "they do these things better in Paris." The probe that touched the bullet in Garibaldi's ancle has, unfortunately, proved a lever to hoist poor Nelaton into the Senate, and thence into professional eclipse and total obscuration.

